# Letter from the Editor in Chief

**DOI:** 10.19102/icrm.2025.16063

**Published:** 2025-06-15

**Authors:** Devi Nair


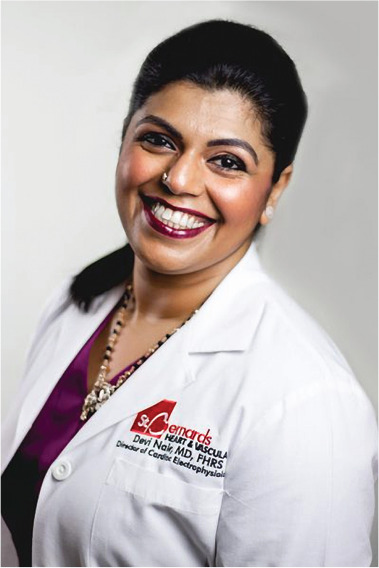


Dear Colleagues,

As we enter the heart of the summer season, we are excited to present the June 2025 issue of *The Journal of Innovations in Cardiac Rhythm Management*, which brings together timely research and clinical insights that reflect the increasingly interconnected world of cardiac electrophysiology.

This month’s issue features original contributions that shine a light on both evolving technologies and enduring challenges in arrhythmia care:

- Racial and sex disparities in stroke prevention among patients with atrial fibrillation (AF) remain unacceptably persistent, as described by Dr. William Tate and colleagues.^1^ Their analysis highlights the need for renewed efforts in improving equitable access to anticoagulation therapy—reminding us that the promise of innovation must be matched by our resolve to deliver it fairly.
- In their meta-analysis, Dr. Rana Ijaz’s team compares remote magnetic navigation and manual catheter navigation for AF ablation, demonstrating the former’s safety advantage and reduced fluoroscopy burden, albeit with longer procedure times.^2^ The findings prompt thoughtful consideration about how we value efficiency, radiation reduction, and long-term outcomes in procedural strategy.
- Dr. Ahmet Taha Sahin and colleagues present a unique case of dual tachycardia—bidirectional ventricular tachycardia and atrial flutter—in a patient with ischemic cardiomyopathy and severe mitral regurgitation.^3^ Their report highlights the diagnostic complexity of their case and the critical role of electrophysiological studies and multidisciplinary care in managing rare arrhythmic presentations.
- Dr. Amit Noheria’s team shares results from a practice-wide shift from traditional right ventricular pacing to left bundle branch area pacing.^4^ Their study demonstrates that, while the newer technique involves longer procedure and fluoroscopy times, it leads to narrower paced QRS durations and fewer adverse remodeling effects without an increase in complication rates, underscoring its viability as a preferred pacing strategy in contemporary care.

## A New Era of Collaboration in Cardiovascular Care

This June also brought renewed attention to the strength of multidisciplinary partnerships, most notably at NY Valves 2025. This year’s meeting introduced the inaugural Left Atrial Appendage Closure (LAAC) Summit, a landmark moment emphasizing the collaborative future of cardiovascular care.

Electrophysiologists, structural cardiologists, imagers, and surgeons came together to explore shared challenges and co-develop strategies for left atrial appendage (LAA) closure—particularly in complex patients who will benefit from cross-specialty insight and procedural expertise. These interactions mark a broader transformation in how rhythm care is delivered: one grounded in teamwork, shared ownership, and common purpose.

Beyond LAA closure, we are seeing an expanding frontier of integration:

- In tricuspid valve interventions, managing existing leads has become a core part of procedural planning. Whether deciding on extraction, alternate pacing, or valve-friendly implantation strategies, these decisions increasingly rely on early collaboration between electrophysiologists, structural operators, and heart failure specialists.
- In cardiac surgery, the role of the electrophysiologist is now central to intraoperative rhythm strategies, including hybrid ablation, mapping, and LAA management. These scenarios demand close coordination before, during, and after surgery to optimize outcomes.

This era of shared expertise is reshaping not just what we do—it is also shaping how we work together to do it better. The success of modern cardiovascular interventions depends as much on collaboration as on innovation.

## Looking Ahead

We hope this issue offers both practical insight and deeper perspective as you care for patients, advance research, and contribute to our ever-evolving field. Thank you for your continued engagement with the journal. We welcome your submissions, your voices, and your partnership as we move forward together.

Warm regards,


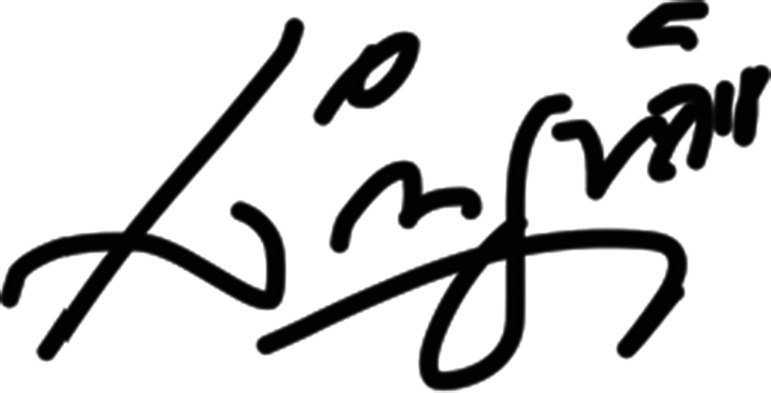


Dr. Devi Nair, md, facc, fhrs

Editor-in-Chief

*The Journal of Innovations in Cardiac Rhythm Management*

Director of the Cardiac Electrophysiology & Research,

St. Bernard’s Heart & Vascular Center, Jonesboro, AR, USA

White River Medical Center, Batesville, AR, USA

President/CEO, Arrhythmia Research Group

Clinical Adjunct Professor, University of Arkansas for Medical Sciences

Governor, Arkansas Chapter of American College of Cardiology

drdgnair@gmail.com
